# Reduced Muscle Strength Can Alter the Impact of Gait Modifications on Knee Cartilage Mechanics

**DOI:** 10.1002/jor.70007

**Published:** 2025-06-27

**Authors:** Joose P. J. Peitola, Amir Esrafilian, Morten B. Simonsen, Michael S. Andersen, Rami K. Korhonen

**Affiliations:** ^1^ Department of Technical Physics University of Eastern Finland Kuopio Finland; ^2^ Department of Materials and Production Aalborg University Aalborg Denmark; ^3^ Center for Mathematical Modeling of Knee Osteoarthritis Aalborg University Aalborg Denmark

## Abstract

Muscle strength can substantially influence knee joint loading, stability, and cartilage biomechanics, all of which are important factors in the onset and progression of knee osteoarthritis (KOA). Noninvasive rehabilitation methods, such as gait modifications, are suggested to effectively alter knee joint loading, potentially helping to prevent or slow KOA progression. However, no studies to date have assessed if reduced lower limb muscle strength can influence the effects of rehabilitation exercises such as gait modifications. This study aimed to reveal how reducing the strength of three lower limb muscle groups (knee extensors, hip abductors, and ankle extensors) impacts the effects of gait modifications (Toe‐out, Toe‐in, and Wide gait) on knee cartilage stresses. We analyzed motion and ground reaction force data from seven healthy male participants using a musculoskeletal‐finite element workflow, where we systematically reduced the isometric strength of the selected muscle groups in the musculoskeletal models. Our findings indicate that reductions in hip abductor and ankle extensor strength had the most pronounced impact on the effects of gait modifications on cartilage mechanics (maximum decrease of 6 percentage points (PP) in the changes of maximum principal stress). Overall, Toe‐out gait was least affected by reduced muscle strength compared to other gait styles, although responses varied greatly between participants (0–14 percentage point differences). This study emphasizes the importance of considering participant‐specific muscle strengths when designing personalized rehabilitation strategies such as gait modifications and provides theoretical insights into optimizing rehabilitation exercises for managing KOA based on a given motion and joint kinetics.

## 1 Introduction

Lower limb muscle strength has been shown to impact knee joint loading, stability, and cartilage mechanics [1, 2, 3, 4, 5, 6], which are all key factors when investigating musculoskeletal diseases such as knee osteoarthritis (KOA) [7, 8, 9]. KOA is a degenerative joint disease causing joint pain and limited functionality due to the deterioration of cartilage and the underlying bone. Currently, no cure exists for KOA. However, various treatment options are available to help manage symptoms, slow disease progression, and improve quality of life. One suggested approach involves using rehabilitation exercises, including gait modification techniques, to reduce loading in the knee joint [10, 11]. Since factors like aging and KOA can lead to reduced muscle strength [7, 9, 12, 13, 14, 15, 16], understanding how altered muscle strength affects cartilage mechanics in different gait modifications can be important for personalized planning of varying rehabilitation strategies. Different gait modifications and exercises may change the location of the loads or result in overloading or underloading of cartilage [5, 17, 18, 19, 20, 21], which have been linked to the potential onset and progression of KOA [10, 12, 22, 23, 24, 25, 26]. Nevertheless, they have not yet been investigated in tandem with pathology‐ and aging‐related reductions in lower limb muscle strength.

With gait modifications, the goal is to change the habitual walking style of an individual in a way that is beneficial for the knee cartilage health, which in most cases means reducing or redistributing the loads in the knee joint. These changes can include modifying the orientation of the feet during walking by voluntarily turning the toes inward or outward, altering the width of the stance, or altering the alignment of the feet with insoles [18, 20, 27]. Another approach to reduce joint loads is to alter muscle coordination, where the goal is to change muscle activations and forces rather than changing the kinematics of the gait [28, 29].

Direct measurement of the loads inside the knee joint is impossible without invasive methods such as instrumented knee implants. Therefore, computational tools such as musculoskeletal (MS) and finite element (FE) modeling have emerged as effective techniques for estimating joint‐ and tissue‐level biomechanics, for instance, joint contact forces (JCF), joint moments, and cartilage stresses and strains [30, 31, 32, 33, 34, 35, 36, 37]. These biomechanical parameters are thought to govern tissue degradation and the onset and progression of KOA [10, 25, 26, 38]. In addition, combined MS and FE modeling approaches have been utilized to estimate the effects of rehabilitation exercises on knee joint loads and cartilage mechanics [18, 39, 40, 41, 42, 43, 44]. Nevertheless, to the best of our knowledge, no studies have yet investigated how the effects of gait modifications on knee cartilage mechanics might be altered when the strengths of the large lower limb muscle groups are reduced as observed in aging populations and people with KOA.

In this study, we investigated the effect of reduced muscle strength on knee cartilage mechanics during habitual gait and three conventional gait modifications. We focused on three muscle groups: knee extensors, which stabilize the knee joint and influence its moments [2, 45]; hip abductors, whose generated moments have been associated with protection against osteoarthritis [46, 47]; and ankle extensors, which primarily contribute to knee joint loads during push‐off phase of gait [2]. Given that these muscle groups affect knee joint loads in distinct ways, we hypothesized that the reduced strength of the hip abductors and knee extensors would lead to increased knee joint loads and consequent cartilage stresses (due to compensatory effects of, e.g., the hip flexors [48]). On the other hand, reducing the strength of the ankle extensors would decrease the loading (due to compensatory effects of, e.g., the hamstring muscles [48]). However, we also hypothesized that the magnitude of these effects would be distinctly different in the different gait styles, and that they would be highly participant‐specific. We utilized previously developed MS‐FE pipelines [18, 43, 49], incorporating participant‐specific kinematics and kinetics from MS analyses of gait modifications as inputs to FE analysis with personalized knee surfaces to estimate stresses on cartilage. First, we analyzed the overall effects of reduced muscle strength on maximum principal stresses across seven participants. We then examined how these effects varied across the different gait modifications, investigating which were most sensitive or resistant to reduced muscle strength in terms of changes in knee cartilage stresses.

## 2 Materials and Methods

### 2.1 MS Modeling

**Figure 1:**
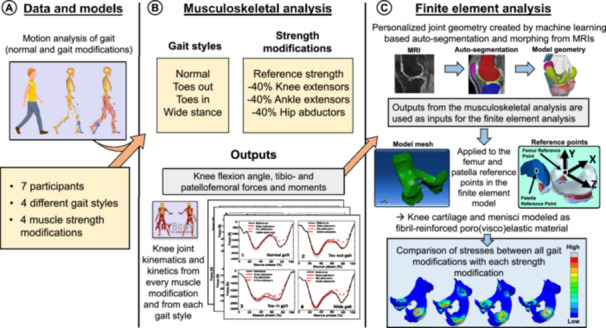
General workflow of the study. Motion and ground reaction force data (A) from seven participants were used in the musculoskeletal modeling analysis (B; AnyBody Modeling System). For each participant, we created four models with different gait styles and four models with different muscle strength modifications. Twelve outputs were analyzed from each participant: knee flexion angle, tibiofemoral forces, tibiofemoral axial rotation and abduction moments, and patellofemoral forces and moments. These outputs were then utilized as inputs in the finite element analysis (C; ABAQUS) which was used to estimate the maximum principal stresses on the medial and lateral tibial cartilage surface during one stance phase of the gait.

Motion capture and ground reaction force data (Figure 1A) were collected from seven healthy male participants (age: 29 ± 6 years, height: 177.7 ± 3.7 cm, weight: 76.3 ± 8.6 kg) at 100 Hz by an 8‐camera Qualisys system (Oqus 300 series, Qualisys, Sweden) and 32 reflective markers. The ground reaction forces and moments were collected at 1000 Hz from two inground force plates (AMTI, USA). The measurements consisted of normal gait and three different gait modifications, consisting of walking with toes slightly turned inward (“Toe‐in gait”), toes slightly turned outward (“Toe‐out gait”), and walking with a wider stance (“Wide gait”). Five gait trials for each gait style with self‐selected walking speeds (Table 1, Table S1) were recorded. The proceedings of the data collection and measurements in this study were confirmed and accepted by the North Denmark Region Committee on Health Research Ethics (Nr. 2022‐000764).

**Table 1:** Maximum, minimum, and mean walking speeds for each gait style.

Gait style	Max speed (m/s)	Min speed (m/s)	Mean speed (m/s)
Normal	1.33	0.98	**1.16** ± **0.14** a,b
Toe‐out	1.20	0.90	**1.05** ± **0.11 (*p* = 0.01)** a
Toe‐in	1.20	0.85	**1.04** ± **0.12 (*p* = 0.03)** b
Wide	1.30	0.91	1.09 ± 0.13

*Note*: Letters “a” and “b” indicate significant differences between gait styles [repeated measures analysis of variance (ANOVA) with Bonferroni corrected post hoc tests].

The creation of the MS models and the analysis were based on our previous studies [18, 21]. In the MS analysis (Figure 1B), the Twente Lower Extremity Model (TLEM v.2.1 [50]) from the AnyBody Managed Model Repository (AMMR 2.5) was utilized in AnyBody (v.7.5.0) Modeling System. The TLEM model was selected for this study since previous research has found it to have a good agreement between experimentally measured (Grand Challenge Competition to Predict In Vivo Knee Loads ‐data) and computationally estimated knee loads [1, 50, 51]. The *xyz*‐method [52] was used for model scaling, whereby the segments are scaled individually in *x*, *y*, and *z* directions. The joint‐to‐joint distances of the model's segments are scaled based on the anthropometric measurements of the participant. Then, the mass and the girth of the segments are scaled based on the participant's overall mass and fat percentage. The fat percentage of the model was estimated from the body mass index with regressions based on the study by Frankenfield et al. [53]. Hill‐type muscle‐tendon units were used, and the muscle strength in the model was determined by the muscle's lean mass and cross‐sectional area, which were obtained by utilizing the *Specific‐Length‐Mass Scaling with fat percent* by Rasmussen et al. [52] as explained for the *xyz*‐scaling. The hip was modeled as a 3‐degree‐of‐freedom (DoF) ball‐and‐a‐socket joint, whereas the knee (tibiofemoral and patellofemoral joints) was modeled as 1 DoF hinge‐type joint, and the ankle as a 2 DoF (plantar/dorsiflexion and inversion/eversion) joint.

Four MS models were created for each participant to investigate how the reduced muscle strengths affected the cartilage mechanics. The first model served as the reference, with no changes made to the muscle strengths. Three additional models were then developed, each with a reduction in the maximum isometric strength of muscle groups that have been shown to play a crucial role in the knee joint loading during walking [2, 4, 22, 54, 55, 56, 57, 58]: the knee extensors (Rectus femoris, Vastus lateralis, Vastus medialis, Vastus intermedius), the hip abductors (Gluteus medius, Gluteus minimus, Tensor fasciae latae), and the ankle extensors (Gastrocnemius, Soleus). This reduction was based on studies showing strength reductions of 15%–60% for knee extensors due to conditions such as KOA [13] or knee surgery [59, 60], and 14%–32% for hip abductors due to KOA [16], and ~40% for ankle extensors due to aging [15]. To ensure a fair comparison and accurately recognize the effects on knee cartilage mechanics, we applied a uniform 40% reduction in the isometric strength across all these selected three muscle groups separately for each model.

Conventional AnyBody pipelines were used to perform inverse kinematics followed by inverse dynamics. Within the inverse dynamics analysis of each gait trial, the muscle forces were estimated using static optimization by solving the following optimization problem [50]:

minimizeG(fM)subject toCf=rfiM≥0,i=1…nM

where *G* is the minimized function, f̅ ^M^ is a vector of muscle forces for a muscle *M*, **C** is a matrix of equation coefficients, r̅ is a vector of external and inertial forces, and *n* ^*M*^ is the total number of muscles in the MS model. In our study, the optimization problem was solved by minimizing the third‐order polynomial muscle recruitment criterion:

(1) $$G=\sum_{i=1}^{n^{M}}{\left(\frac{f_{i}^{M}}{N_{i}}\right)}^{3}$$

where *N*_*i*_ is the isometric strength in the muscle model [61].

### 2.2 FE Modeling

#### 2.2.1 Material Properties and Meshing

The knee joint FE models were developed in ABAQUS (version 6.23, Dassault Systѐmes, US) based on the study by Esrafilian et al. [62]. The personalized joint geometries were created from participants’ MRIs (Scanner: Signa HDxt 3.0 T; Sequence: 3D SE, Sag DP Cube FS; Slice thickness: 0.8 mm) by utilizing a machine learning‐based auto‐segmentation and morphing method [62], and then cartilage and menisci were meshed [18] (HyperMesh 2019) with hexahedral elements with pore pressure (element type C3D8P). The anterior and posterior cruciate ligaments (ACL) [63], lateral and medial collateral ligaments [63], lateral and medial patellofemoral ligaments [64], patellar tendon [65], and menisci horn attachments [66] were also extracted from the participants’ MRIs and modeled as nonlinear spring bundles. The femoral, patellar, and tibial cartilages were modeled as fibril‐reinforced poroviscoelastic materials and menisci as fibril‐reinforced poroelastic materials [36, 67, 68]. The mesh independence of the cartilage mechanics has been assured in our previous studies [69, 70]. More detailed information about the material parameters can be found in the Supporting Information S1.

#### 2.2.2 Boundary Conditions, Loading, and FE Analysis

The FE models included 6‐DoF tibiofemoral and 6‐DoF patellofemoral joints, with the knee coordinate system fixed to the tibia. In full extension, the origin was positioned at the center of the distal femoral head, between the medial and lateral femoral condyles. The positive directions of the axes were defined as +*x* for the posterior direction, +*y* for the upward direction, and +*z* for the lateral direction (Figure 1C). The alignment between the MS knee joint model and the FE knee joint model was accomplished via a rigid‐body transformation. The models’ coordinate systems were aligned by merging them at corresponding anatomical landmarks, such as the centers of the medial and lateral femoral epicondyles, the medial and lateral articular facets of the patella, and the medial and lateral intercondylar tubercles of the proximal tibia. These key points were identified from both the MS and FE models using their stereolithography files in MeshLab [71].

Femoral and patellar reference points were defined in the FE model based on the origins of their coordinate systems in the MS model (Figure 1C). The femoral and patellar cartilage, along with the nodes on the cartilage–subchondral bone interface, were coupled to these reference points accordingly. The outputs from the MS analysis (provided below), used as inputs for the FE models, were applied to the femoral and patellar reference points, while the base of the tibia was fixed in all FE models.

The inputs for the FE analysis were: (1) the knee flexion angle, and (2) the net forces and moments acting on the distal femur due to inertial, gravitational, muscle, and hip reaction forces, all applied to the femoral reference point. In addition, inputs applied to the patellar reference points included (3) the net forces and moments from inertia, gravity, and the quadriceps muscles. Four FE models per participant, each using inputs from the four different MS models and an average from five gait trials for each gait style (one habitual gait and three gait modifications) throughout the stance phase (heel strike to toe‐off) were generated and run in the soil consolidation solver of ABAQUS. An example of the loading inputs for one participant is provided in Figure S1.

Certain tissues, such as the knee capsule and skin, which influence secondary knee kinematics, were not explicitly modeled in the FE simulations of this study. The omission of these tissues can often result in excessive secondary knee movements [42]. To account for this, previous studies have typically downscaled inputs or applied kinematic constraints to replicate the effects of these tissues and ensure the FE models achieve convergence [37, 42, 68]. Exploiting our previous studies [37, 42, 49, 68], we used passive connector elements to represent the effect of these omitted tissues in restraining knee joint movements. The connectors were implemented based on earlier studies using multiple DoFs [19, 72], with their stiffness values fine‐tuned to ensure consistency in the secondary knee kinematics during gait with existing literature [73, 74, 75]. Specifically, the anteroposterior and mediolateral translations in the tibiofemoral joint were restrained with connector stiffness values of 0.5 N/mm, while internal and external rotations were constrained with a 1.2 Nm/degree stiffness. A stiffness of 0.2 N/mm limited the mediolateral translation of the patella, and all patellar rotational degrees of freedom were restricted with stiffness values of 0.9 Nm/degree.

The tibial cartilage mechanical responses investigated in this study were represented by the mean values of the upper quartile of maximum principal stress from the superficial layer of the cartilage. This includes the top 25% of elements on the tibial cartilage contact area that experienced the highest stress magnitudes. With this choice, we wanted to ensure that potentially outlying and unreasonably high stress values for some individual elements would not substantially affect the resulting quantitative values for stress. This choice has also been used in previous studies regarding FE modeling [41, 76].

We first quantified the effect of reduced muscle strength on maximum principal stresses for each gait style and participants. This was achieved by calculating the percentage change in the stresses %Change_Gait_ in each of the four gait styles between the reduced strength models and the reference (unmodified strength) model:

(2) $${\%\text{Change}}_{\text{Gait}}=│\frac{\sigma_{\text{Reduced strength}}-\sigma_{\text{Ref}}}{\sigma_{\text{Ref}}}│\cdot 100\%,$$

where $\sigma_{\text{Reduced strength}}$ is the maximum principal stress from the model with reduced strength, $\sigma_{\text{Ref}}$ is the maximum principal stress from the reference model, and %Change_Gait_ is denoted as ${\%\text{Change}\sigma }_{\text{Modified}}$ for the three gait modifications and %Change_Normal_ for the normal gait. Next, to examine how these percentage changes varied between the gait modifications, we calculated the percentage point difference (PP_Difference_) by subtracting the absolute value of percentage change in the stresses in normal gait from the absolute value of percentage change in each gait modification:

(3) $${\text{PP}}_{\text{Difference}}={\%\text{Change}}_{\text{Modified}}–{\%\text{Change}}_{\text{Normal}}$$

In addition to calculating these differences for each participant, an average percentage point difference from all seven participants was calculated for the maximum principal stresses for each gait modification. These calculations allowed us to analyze the sensitivity of the effects of gait modifications to the reduced lower limb muscle strengths; negative/positive PP_Difference_ indicates smaller/greater difference in stresses in the modified gait compared to normal gait when muscle strength is reduced (i.e., cartilage mechanical stress response in certain gait style is less/more sensitive to selected strength reduction, respectively).

### 2.3 Statistical Analysis

Comparisons between the participants’ walking speeds were analyzed with repeated measures analysis of variance with Bonferroni corrected post hoc tests. A paired *t*‐test was used to compare differences between the peak stress values from the reference model and the models with the reduced muscle strengths. A significance level of *α* < 0.05 was chosen for all statistical tests.

## 3 Results

**Figure 2:**
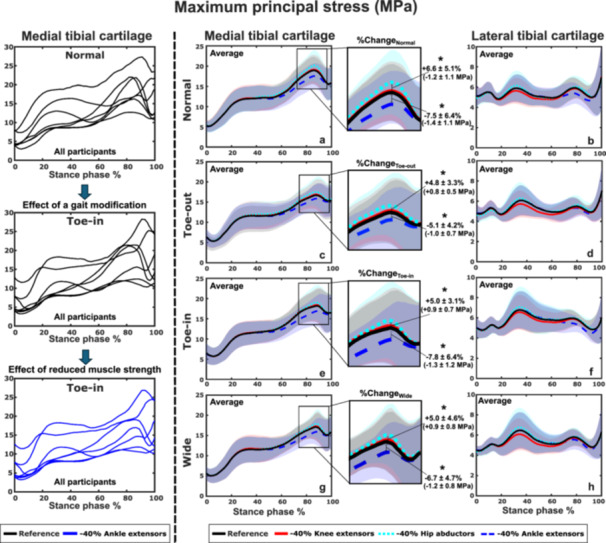
The average effect of reduced muscle strength on maximum principal stresses in the medial (a, c, e, g) and lateral (b, d, f, h) tibial cartilage across all seven participants walking with normal and modified gait. Plots show the mean change in stress (±95% confidence intervals represented by the shaded areas) between the reduced strength and reference (unmodified strength) models. The “*” symbol represents statistical significance (*p* < 0.05). Overall, in the medial tibial cartilage, reduced hip abductor strength was associated with increased stresses while reduced ankle extensor strength was associated with decreased stresses in all gait styles.

The reduced muscle strengths had similar effects on the average maximum principal stress across all the gait styles in both medial and lateral tibial cartilage (Figure 2). These effects were more pronounced in the medial tibial cartilage (Figure 2a,e,c,g) than in the lateral tibial cartilage (Figure 2b,d,f,h). Reducing the hip abductors’ strength significantly (*p* < 0.05) increased the peak stress values of the medial tibial cartilage in all the gait styles, with the greatest increase observed in normal gait (6.6% ± 5.1%) at ~80% of the stance phase. Reducing the ankle extensors’ strength significantly decreased the stress in all gait styles (%maximum of −7.8% ± 3.1% in Toe‐in gait) at the same time point. Reducing the knee extensors’ strength produced no significant differences in the peak cartilage stresses.

**Figure 3:**
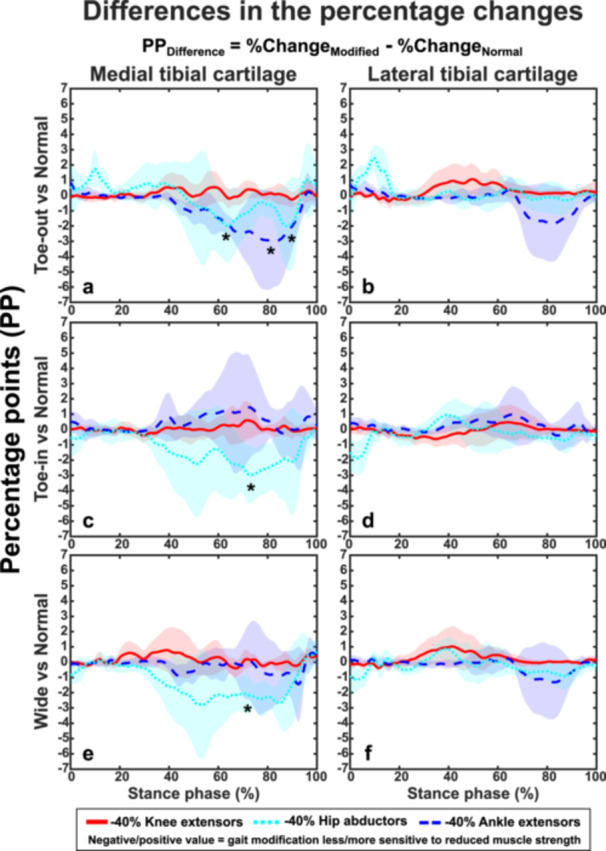
Sensitivity of the effects of gait modifications (on cartilage mechanical stress responses) to the reduced lower limb muscle strengths. Average difference in the absolute values of maximum principal stress percentage changes from all seven participants (relative to the reference (unmodified muscle strength) model represented by the value zero) between different gait modifications and normal gait. The “*” symbol represents statistical significance (*p* < 0.05) at a certain time point. The stresses during the Toe‐out gait were significantly less sensitive to the reduced ankle extensor strength (i.e., negative PP_difference_, smaller stress difference in Toe‐out gait vs. normal gait when ankle extensor strength was reduced) in medial (a) tibial cartilage, whereas the stresses during the Toe‐in and Wide gait were significantly less sensitive to the reduced hip abductor strength in the medial tibial cartilage (c, e). No statistically significant differences were observed in the lateral tibial cartilage (b, d, f).

In Toe‐out gait, compared to normal gait, reducing the ankle extensors’ strength produced an average of 3.0 ± 2.5 PP smaller change (*p* < 0.05) to the medial tibial cartilage stresses at ~80% of the stance phase (Figure 3a). Similarly, reducing the hip abductors’ strength produced an average of 2.0 ± 1.8 PP smaller change (*p* < 0.05) to the medial tibial cartilage stresses at ~60% and ~90% of the stance phase (Figure 3a). In Toe‐in and Wide gait, compared to normal gait, reducing the hip abductors’ strength produced 3.0 ± 2.8 PP and 2.2 ± 1.0 PP smaller changes (*p* < 0.05) to the medial tibial cartilage stresses at ~70% of the stance phase (Figure 3c,e). Reducing the knee extensors’ strength produced small and similar (*p* > 0.05) effect to the cartilage stresses in all gait modifications.

**Figure 4:**
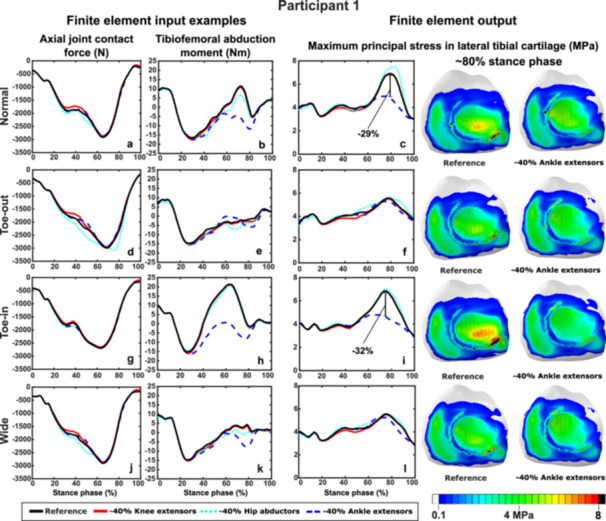
Estimated axial joint contact forces, abduction moments, and maximum principal stresses for Participant 1 during each gait style. Reducing the ankle extensor muscle strength led to lower abduction moments and maximum principal stress mainly during normal gait (b, c) and the Toe‐in gait (h, i).

To highlight the personalized effects of the reduced muscle strengths, we selected two participants who had substantial differences in the resulting knee cartilage stresses. Regarding participant‐specific responses for Participant 1, the most notable changes in the abduction moments (Figure 4b,e,h,k) and maximum principal stresses (Figure 4c,f,I,l) were observed with the reduced strength of the ankle extensors at early terminal stance (~60%–70% stance phase), specifically in Normal and Toe‐in gait. In normal gait, reducing the strength of the ankle extensors decreased the tibiofemoral abduction moments (applied as an input to the FE model) from 10 Nm to −5 Nm and the maximum principal stress from 7 to 5 MPa (Figure 4b,c). In contrast, in the Toe‐in gait, the abduction moment and maximum principal stress were reduced from 20 to 0 Nm and 7 to 4.5 MPa, respectively (Figure 4h,i).

**Figure 5:**
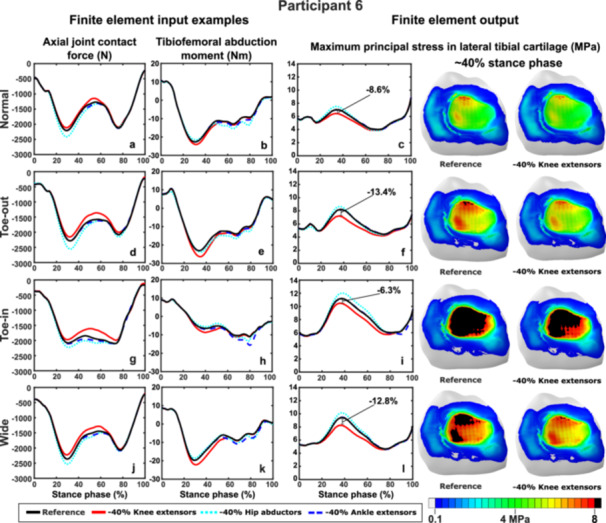
Estimated axial joint contact forces, abduction moments, and maximum principal stresses for Participant 6 during each gait style. Reducing the knee extensor muscle strength decreased the joint contact forces (a, b, g, j), increased the tibiofemoral adduction moments (b, e, h, k) and decreased the maximum principal stresses in the lateral tibial cartilage in all gait styles.

For Participant 6, the most notable changes were the decreased axial JCFs (Figure 5a,d,g,j) and maximum principal stresses (Figure 5c,f,i,l) observed with the reduced strength of the knee extensors in all gait styles at mid stance to terminal stance (30%–70% of the stance phase). In contrast, the reduced strength of the hip abductors increased the JCFs and maximum principal stresses during mid‐stance (~35%–40% of the stance phase) in all gait styles.

**Figure 6:**
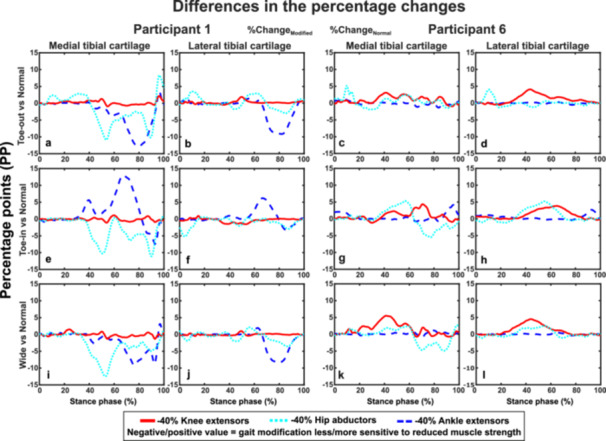
Differences in the maximum principal stress changes (relative to the Reference model represented by the zero line) for Participants 1 and 6 due to reduced muscle strength during each gait modification compared to normal gait. For Participant 1, the stresses during Toe‐in gait (e, f) were more sensitive to reduced ankle extensor strength and less sensitive during the other gait modifications. For Participant 6, the stresses appeared to be more sensitive to reduced knee extensor strength across all gait modifications.

Regarding the participant‐specific sensitivity of gait modifications to reduced muscle strength for Participant 1, the most notable differences were observed in the medial tibial cartilage with reduced strength of the hip abductors and ankle extensors in all gait modifications (Figure 6a,e,i). The reduced strength of the hip abductors resulted in ~11–13 PP smaller change to the maximum principal stress in the medial tibial cartilage at mid‐stance (~50% of the stance phase) in all gait modifications compared to normal gait. This was also the case with the reduced strength of the ankle extensors in Toe‐out and Wide gait but not in Toe‐in gait, where the change in the stress was increased by ~13 PP compared to normal gait.

For Participant 6, the reduced strength of the knee extensors was associated with ~5 PP greater changes in the stress compared to normal gait in both medial (Figure 6c,g,k) and lateral (Figure 6d,h,l) tibial cartilage in each gait modification at mid‐stance to terminal stance (~40%–70% of the stance phase). The reduced strength of the ankle extensor resulted in negligible differences in the stresses. In contrast, the model with reduced strength of the hip abductors estimated greater changes (~5 PP) in Toe‐in gait at mid‐stance (~50% of the stance phase) but not in other gait modifications compared to normal gait.

**Figure 7:**
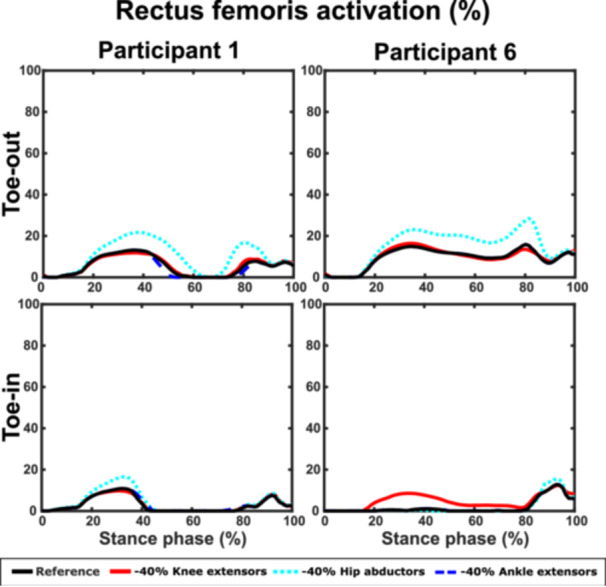
Activations of rectus femoris muscle in Toe‐out and Toe‐in gait with all the muscle strength modifications for Participants 1 and 6. For Participant 1, the activation increased with reduced hip abductor muscle strength in both gait styles, whereas with Participant 6 the activation only increased in Toe‐out gait. For Participant 6, the reduced strength of the knee extensors caused a notable increase in the muscle activation during Toe‐in gait, which was not present with Participant 1.

The activation level of the rectus femoris muscle increased in each gait style with reduced hip abductor strength for both Participants 1 and 6 except, for the Toe‐in gait for Participant 6 (Figures 7 and S2). In addition, for Participant 6, the rectus femoris muscle showed increased activity with reduced knee extensor strength in normal, Toe‐in, and wide gait.

## 4 Discussion

In this study, we investigated how reduced lower limb muscle strengths affect the impact of gait modifications on knee cartilage mechanics. We used an MS‐FE analysis pipeline incorporating participant‐specific motion analysis from habitual gait and three gait modifications. We modeled strength reduction in three large lower limb muscle groups relevant to conditions such as KOA, ACL surgery, total knee arthroplasty, and aging. In accordance with our hypothesis, the reduced muscle strength altered the cartilage mechanical responses and the effects of gait modifications in distinct ways. On average, the reduced strength of the ankle extensors and hip abductors had the greatest impact on tibial cartilage stress response. Supporting our hypothesis, reducing ankle extensor strength decreased the stresses, whereas reducing hip abductor strength increased the stresses (Figure 2). Strength reduction in these same muscle groups distinctly varied the effects of gait modifications: Toe‐out gait was less affected by the reduced strength of the ankle extensors (negative values in Figure 3a,b) compared to Toe‐in (Figure 3c,d) and Wide gait (Figure 3e,f), which in turn were less sensitive to the reduced strength of the hip abductors. Importantly, these effects varied greatly between individuals and their respective gait styles (Figures 4, 5, 6). This highlights the suitability of the developed modeling pipeline to find optimal participant‐specific gait modification, now also in individuals with reduced muscle strength, to alter cartilage mechanical stresses and lower the risk of KOA development.

The reduced strength of the ankle extensors had the greatest effect on the maximum principal stress within the tibial cartilage in all gait styles at the time of the second ground reaction force peak (~80% of the stance phase). This result is in accordance with previous studies where the ankle extensors, especially the gastrocnemii, are most active during the terminal stance [2, 77, 78]. Reduced hip abductor strength has also been suggested to increase knee joint loads during walking, especially in the medial compartment [46, 47, 79]. This was also the case in our study, where the reduced hip abductor strength increased maximum principal stresses at the late stance phase. Although quadriceps strengthening is commonly recommended in exercise therapy for KOA [24, 80], the present study indicates that reduced quadriceps strength has a limited effect on the average maximum principal stresses on tibial cartilage. One factor explaining this could be that, in reality, the participants’ gait and knee joint kinematics would change if they had reduced muscle strengths [80, 81]. However, in our study, the kinematics of the participants remained the same throughout the MS models since we only reduced the isometric strengths in the models.

We also investigated how reduced muscle strength affected the maximum principal stress across the different gait modifications compared to normal gait (Figure 3). Interestingly, in Toe‐out gait, reduced ankle extensor strength resulted in smaller stress differences compared to Normal gait (~3 PP, Figure 3a,b), while this was not the case for Toe‐in (Figure 3c,d) and Wide gait (Figure 3e,f). Conversely, Toe‐in and Wide gaits were less sensitive to reduced hip abductor strength than Toe‐out gait. These findings align with substantial changes in muscle activations across the different gait modifications (Figures 7 and S1 and S2), especially in the knee and ankle extensors. This further indicates that muscle strength plays a key role in the effects of gait modifications on cartilage mechanics. In addition, Toe‐out gait appears to be the least sensitive to changes in lower limb muscle strength compared to other gait modifications. Since gait modifications are used as rehabilitation methods for KOA patients [10, 11, 21], who may experience muscle weakness [47, 56, 80], considering participant‐specific strengths is crucial when planning rehabilitation for this patient group.

Some differences were also observed in the walking speeds between the gait styles (Table 1). Since faster walking speed has been suggested to increase the knee joint loads [82], this may be one of the reasons why, for example, in Normal gait the stresses were overall higher than in Toe‐out gait.

In addition to analyzing the average cartilage stress results, we highlighted participant‐specific outcomes for two selected participants, showing notable differences in the effects of reduced muscle strengths and gait modifications on cartilage mechanics (Figures 4, 5, 6). For Participant 1, reduced ankle extensor strength was associated with significant changes in tibiofemoral abduction moments (an input to the FE model) and maximum principal stresses during both normal gait (Figure 4b,c) and Toe‐in gait (Figure 4h,i) at the second ground reaction force peak. The reduced strength of the ankle extensors and hip abductors also caused substantial differences (~10–15 PP) in the medial tibial cartilage during each gait modification. In contrast, for Participant 6, reduced ankle extensor strength had minimal effects on knee axial contact forces, tibiofemoral abduction moments, and maximum principal stresses (Figure 5). However, reduced knee extensor strength for Participant 6 resulted in the most notable impacts on knee contact forces, moments, and stresses in each gait style, with greater differences (~4–5 PP) in stresses during gait modifications compared to Normal gait. This emphasizes the importance of muscle strength when selecting rehabilitation methods and demonstrates how gait modifications can differently affect individuals. It also shows how reduced muscle strengths can alter these effects on cartilage mechanics.

The differences in the muscle activation levels (Figures 7 and S2 and S3) for the two selected participants could explain the altered effects of the reduced muscle strengths on cartilage mechanics during different gait modifications. The average (Figure 3) and the participant‐specific results (Figures 4, 5, 6) suggest that muscle activation levels change differently when the muscle strength is reduced [83, 84] and it is dependent on the gait style. This result is not surprising since every individual has a unique way of walking [85, 86], and modifying it in a certain way will require personalized muscle contributions. For example, an individual with a slight toe‐out position or naturally pronated feet may find it easier to perform a Toe‐out gait compared to another who naturally walks with toes pointed forward or inward or have naturally supinated feet. Therefore, the muscles’ activations of the first individual would likely need to change less compared to the second individual.

To further evaluate inter‐participant variability in the different gait styles, we conducted an additional analysis using one‐dimensional Statistical Parametric Mapping in MATLAB (SPM1D) across all the gait styles for hip, knee, and ankle flexion kinematic waveforms (Figure S2). Similar kinematic patterns were observed for all participants, though some inter‐participant differences were observed (*p* < 0.05). These differences, along with the variation in walking speeds (Table S2), may explain some of the participant‐specific results in the cartilage mechanics.

This study was not without limitations. First, we used generic, linearly scaled MS models validated in previous research, such as the Grand Challenge Competition to Predict In Vivo Knee Loads [51, 87]. We acknowledge uncertainties in these models, including bone geometries, joint alignments, and muscle insertion points. We recognize that personalizing the MS models could help reduce such uncertainties [21, 88, 89]. However, since the model validation, such as joint contact forces, against experiments of living subjects was not feasible in our study, we did not pursue personalizing the MS models. In addition, the use of linearly scaled models is more widely applicable and feasible to researchers at different levels of expertise. In addition, the 1 DoF knee joint MS models used in this study may present limitations as it has been shown that joint constraints can affect joint loading [90]. However, prior research [42] has demonstrated that FE models driven by 1 DoF MS models provide estimates of secondary knee kinematics and cartilage biomechanics comparable to more complex 12 DoF MS models. Lastly, the experimental inputs were consistent across all models and did not account for the kinematic and kinetic changes that could arise from the reduced muscle strengths. Therefore, the effects of the potentially altered kinematics on the joint loads and cartilage mechanics are neglected, which could limit the accuracy of the estimated stresses between the different strength conditions.

Although muscle strength is affected differently due to conditions such as KOA [13, 47, 57], ACL reconstruction surgery [60, 91], and knee replacement [59, 92], or because of aging [15], we applied a uniform 40% reduction in isometric strength across the three muscle groups. Although previously reported levels of strength changes are not the same for all the studied muscles [14, 15, 16, 59, 60], this approach allowed us to fairly compare the effects of reduced strength on cartilage mechanics across all muscle groups. However, we do acknowledge that different levels of strength reductions could result in changes in muscle activations, and that aging could lead to altered kinematics and muscle coordination [93, 94], thus altering the resulting cartilage mechanics. For a more comprehensive analysis of the effects of muscle strength on cartilage stresses in different gait modifications, future research could consider using forward simulation methods [95] to allow deviations from the given motion when muscle strength is reduced. In addition, increasing the number of participants, incorporating a wider range of muscle strength levels, and integrating personalized muscle‐tendon parameters could further enhance the applicability of the findings.

In conclusion, our study suggests that rehabilitation methods like gait modifications theoretically affect knee mechanics differently when lower limb muscle strength is reduced. Certain gait modifications may be less (Toe‐out gait) or more (Toe‐in gait) sensitive to reductions in muscle strength (e.g., reduced ankle extensor strength) than others, meaning uncertainties in MS models about muscle strengths would have less/more impact on outcomes depending on the gait modification. However, these findings are highly participant‐specific and should be considered when designing personalized rehabilitation strategies. As our results are based on assumption of unchanged ground reaction forces and joint kinematics, they provide theoretical insights into computational modeling‐assisted rehabilitation tailoring and future studies evaluating the impacts of these interventions on cartilage health.

## Author Contributions

**Joose P. J. Peitola:** conceptualization, musculoskeletal modeling, finite element modeling, interpretation of the data, writing – original draft. **Amir Esrafilian:** conceptualization, supervision, assisting with finite element modeling, review and editing. **Morten B. Simonsen:** conceptualization, data collection, assisting with musculoskeletal modeling, review and editing. **Michael S. Andersen and Rami K. Korhonen:** conceptualization, supervision, funding acquisition, review and editing.

## Conflicts of Interest

The authors declare no conflicts of interest.

## Supporting information

Supp Material.
