# Supplementary material for: Reduced Muscle Strength Can Alter the Impact of Gait Modifications on Knee Cartilage Mechanics
Source: J Orthop Res. 2025 Jun 27;43(9):1566–80. doi: 10.1002/jor.70007 (PMC12329646; doi:10.1002/jor.70007)
Supplement: Supplementary file 1 — Supp Material. [file JOR-43-1566-s001.docx]

**Supplementary material for:**

**Reduced muscle strength can alter the impact of gait modifications on knee cartilage mechanics**

Joose P. J. Peitola^1^, Amir Esrafilian^1^, Morten B. Simonsen^2,3^, Michael S. Andersen^2,3^, Rami K. Korhonen^1^

^1^Department of Technical Physics, University of Eastern Finland, Kuopio, Finland

^2^Department of Materials and Production, Aalborg University, Aalborg, Denmark

^3^Center for Mathematical Modeling of Knee Osteoarthritis, Aalborg University, Aalborg, Denmark

**Corresponding author’s email: joose.peitola@uef.fi**

**2. MATERIALS AND METHODS**

***2.1 MS Modeling***

**Table S1.** Walking speeds of each participant in each gait style.

| **Participant** | **Gait style** | **Mean speed (m/s)** | **Standard deviation (m/s)** |
| --- | --- | --- | --- |
| 1 | Normal | 0.98 | 0.03 |
| 2 | Normal | 1.17 | 0.02 |
| 3 | Normal | 1.33 | 0.02 |
| 4 | Normal | 1.07 | 0.02 |
| 5 | Normal | 1.18 | 0.06 |
| 6 | Normal | 1.32 | 0.03 |
| 7 | Normal | 1.05 | 0.03 |
| 1 | Toe-out | 0.93 | 0.02 |
| 2 | Toe-out | 1.03 | 0.04 |
| 3 | Toe-out | 1.16 | 0.04 |
| 4 | Toe-out | 0.90 | 0.02 |
| 5 | Toe-out | 1.12 | 0.06 |
| 6 | Toe-out | 1.20 | 0.03 |
| 7 | Toe-out | 1.01 | 0.05 |
| 1 | Toe-in | 0.95 | 0.04 |
| 2 | Toe-in | 1.02 | 0.02 |
| 3 | Toe-in | 1.20 | 0.03 |
| 4 | Toe-in | 0.85 | 0.04 |
| 5 | Toe-in | 1.15 | 0.04 |
| 6 | Toe-in | 1.14 | 0.02 |
| 7 | Toe-in | 0.96 | 0.03 |
| 1 | Wide | 0.94 | 0.01 |
| 2 | Wide | 1.04 | 0.03 |
| 3 | Wide | 1.20 | 0.02 |
| 4 | Wide | 0.91 | 0.04 |
| 5 | Wide | 1.18 | 0.06 |
| 6 | Wide | 1.30 | 0.02 |
| 7 | Wide | 1.04 | 0.03 |

***2.2 FE Modeling***

2.2.1 *MATERIAL PROPERTIES AND MESHING*

**Table S2.** Material parameters for the knee joint cartilage and menisci

| Material parameter | Description | Femoral cartilage | Tibial  cartilage | Patellar cartilage | Menisci |
| --- | --- | --- | --- | --- | --- |
| E_0_ (MPa) | Initial modulus of  Collagen fibrils | 0.92 [1] | 0.18 [1] | 1.88 [1] | 28 [2] |
| E_ɛ_ (MPa) | Strain-dependent modulus of Collagen fibrils | 150 [1] | 23.6 [1] | 597 [1] | - |
| *ɳ* (MPa·s) | Damping coefficient of Collagen fibrils | 1062 [3] | 1062 [3] | 1062 [3] | - |
| C (-) | Ratio of primary and secondary fibrils | 12.16 [3] | 12.16 [3] | 12.16 [3] | 12.16 [3] |
| E_nf_ (MPa) | Young’s modulus of  Non-fibrillar matrix | 0.215 [1] | 0.106 [1] | 0.505 [1] | 0.5 [2] |
| *v*_nf_ (-) | Poisson’s ratio of  Non-fibrillar matrix | 0.15 [3] | 0.15 [3] | 0.15 [3] | 0.36 [2] |
| k_0_ $\left( \frac{\boldsymbol{m}^{\boldsymbol{4}}}{\boldsymbol{N\cdot s}}\boldsymbol{\cdot}\mathbf{10}^{\boldsymbol{-15}} \right)$ | Initial permeability | 6 [1] | 18 [1] | 1.9 [1] | 1.25 [4] |
| M (-) | Material constant for strain-dependent permeability | 5.09 [1] | 15.64 [1] | 15.93 [1] | 5.09 [1] |
| n_f,eq_ (-) | Fluid fraction in equilibrium  with normalized depth *d*_n_ | 0.85−0.15*d*_n_ [5] | 0.85−0.15*d*_n_ [5] | 0.85−0.15*d*_n_ [5] | 0.72 [5] |

Ligaments and tendons were modeled as spring bundles to have sufficient accuracy in the estimated parameters while keeping the computational demand reasonable [6]. Ligament and tendon insertion points were segmented from the template MRIs. Non-linear spring bundles were used to replicate the Anterior cruciate ligament (ACL, 59 springs), posterior cruciate ligament (PCL, 102 springs), lateral collateral ligament (LCL, 22 springs), and medial collateral ligament (MCL, 15 springs). Utilizing a bundle of springs provides the ligament model with compression-tension nonlinearity with different properties along and perpendicular to the fibril/spring directions. The slack, toe, and linear regions of the ligaments were formulated according to the study by Blankevoort et al. [7] as:

$f_{s}=\left\{ \begin{aligned} &0 &&,\epsilon_{s}<0 \\ &\frac{1}{4}K_{s}\epsilon_{s}^{2}/\epsilon_{l} &&,0\leq\epsilon_{s}\leq2\epsilon_{l} \\ &K_{s}\left( \epsilon_{s}-\epsilon_{l} \right) &&,\epsilon_{s}\geq2\epsilon_{l} \end{aligned} \right.$ (1)

where $f_{s}$ is the tensile force in each ligament element, $K_{s}$ is the ligament stiffness [7], $\epsilon_{l}$ is the end of the toe region and it was set to 0.03 [8], and $\epsilon_{s}$ is the current strain in the ligament.

The medial and lateral patellofemoral ligaments (MPFL and LPFL, respectively) were modeled using linear spring bundles with no compressive resistance. The spring stiffness (i.e. as the bundle) was defined as 15.9 N/mm for MPFL and 11.7 N/mm for LPFL [9]. Menisci horn attachments were modeled as linear spring bundles with a total stiffness of 336 N/mm and 381 N/mm for the anterior and posterior sides, respectively [10]. Similarly, the patellar tendon was represented by two springs (no resistance in compression) with a total spring constant equal to 545 N/mm [11].

2.2.2 *BOUNDARY CONDITIONS, LOADING AND FE ANALYSIS*

The complete list of the MS model outputs used as inputs in the FE analysis was knee flexion angle; tibiofemoral forces in the axial, mediolateral and anteroposterior directions and resultant tibiofemoral internal-external rotation moment and abduction-adduction moment, which were all summed up from the muscle forces, hip reaction forces, gravity and inertia, and applied to the femoral reference point; patellofemoral forces in the axial, mediolateral and anteroposterior directions, and patellofemoral rotation moments in flexion-extension, mediolateral, and anteroposterior directions, which were all summed up from the inertia, gravity and quadriceps muscle forces, and applied to the patellar reference point. An example of this set of inputs is illustrated in Fig. S1.

**
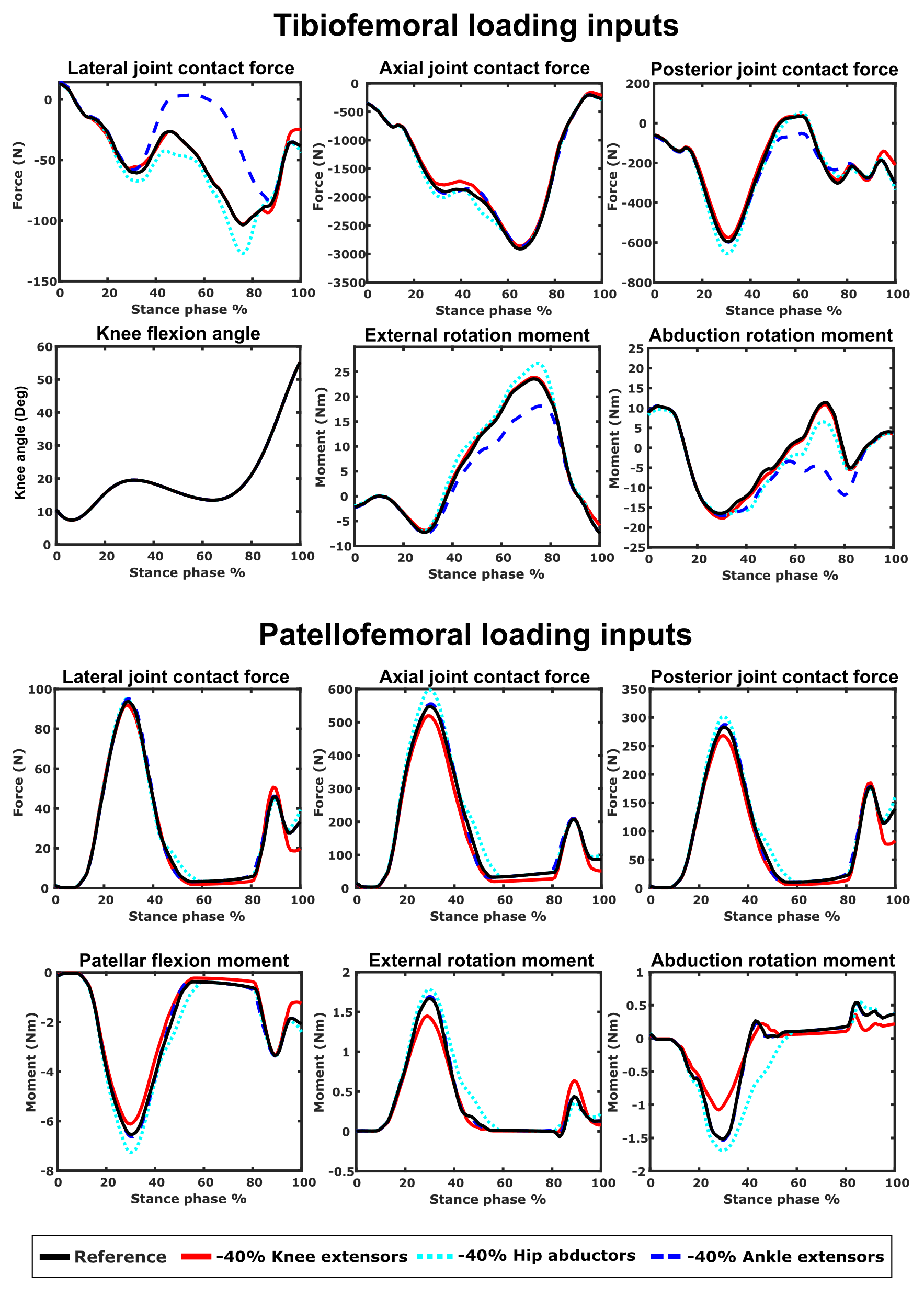
**

**Fig. S1**. An example from one participant of the outputs of the musculoskeletal analysis (AnyBody Modeling System) which were utilized as inputs in the finite element analysis (ABAQUS). The inputs were the knee flexion angle, tibiofemoral joint contact forces and moments and patellofemoral joint contact forces and moments.

**3. RESULTS**

**
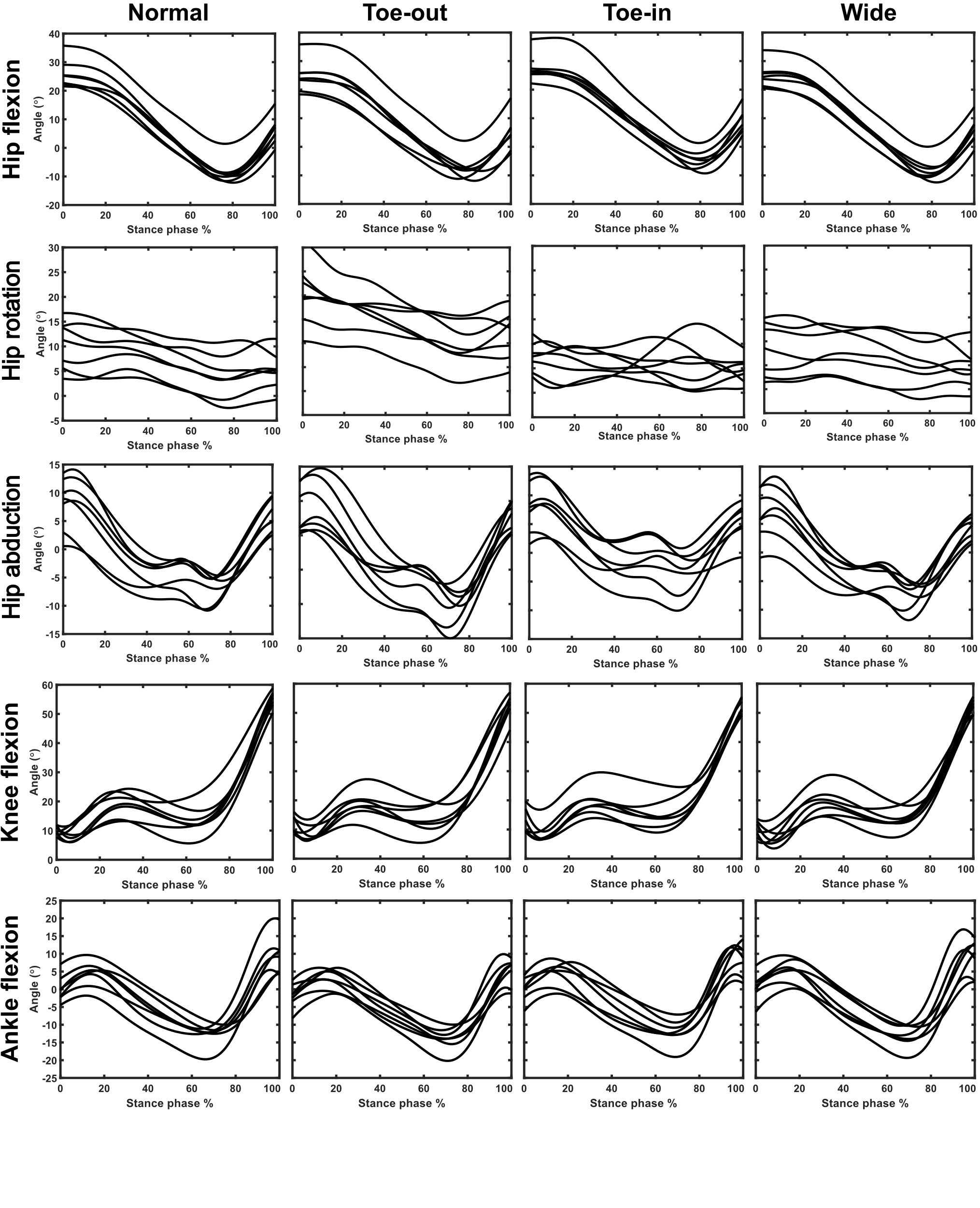
**

**Fig S2**. Kinematic waveforms of hip flexion, hip rotation, hip abduction, and knee and ankle (plantar) flexion values from all seven participants in each gait style.

**Fig. S3**. Activations of rectus femoris and vastus muscles in each gait style with all the muscle strength modifications for Participants 1 and 6. For Participant 1, the activation of the rectus femoris muscle increased with reduced hip abductor muscle strength in all gait styles, whereas with Participant 6 the activation only increased in Normal and Toe-out gait. The vastus muscle had increased activation levels in each gait style with reduced knee extensor muscle strength for both participants. For Participant 6, this was also the case with the rectus femoris muscle except in Toe-out gait.


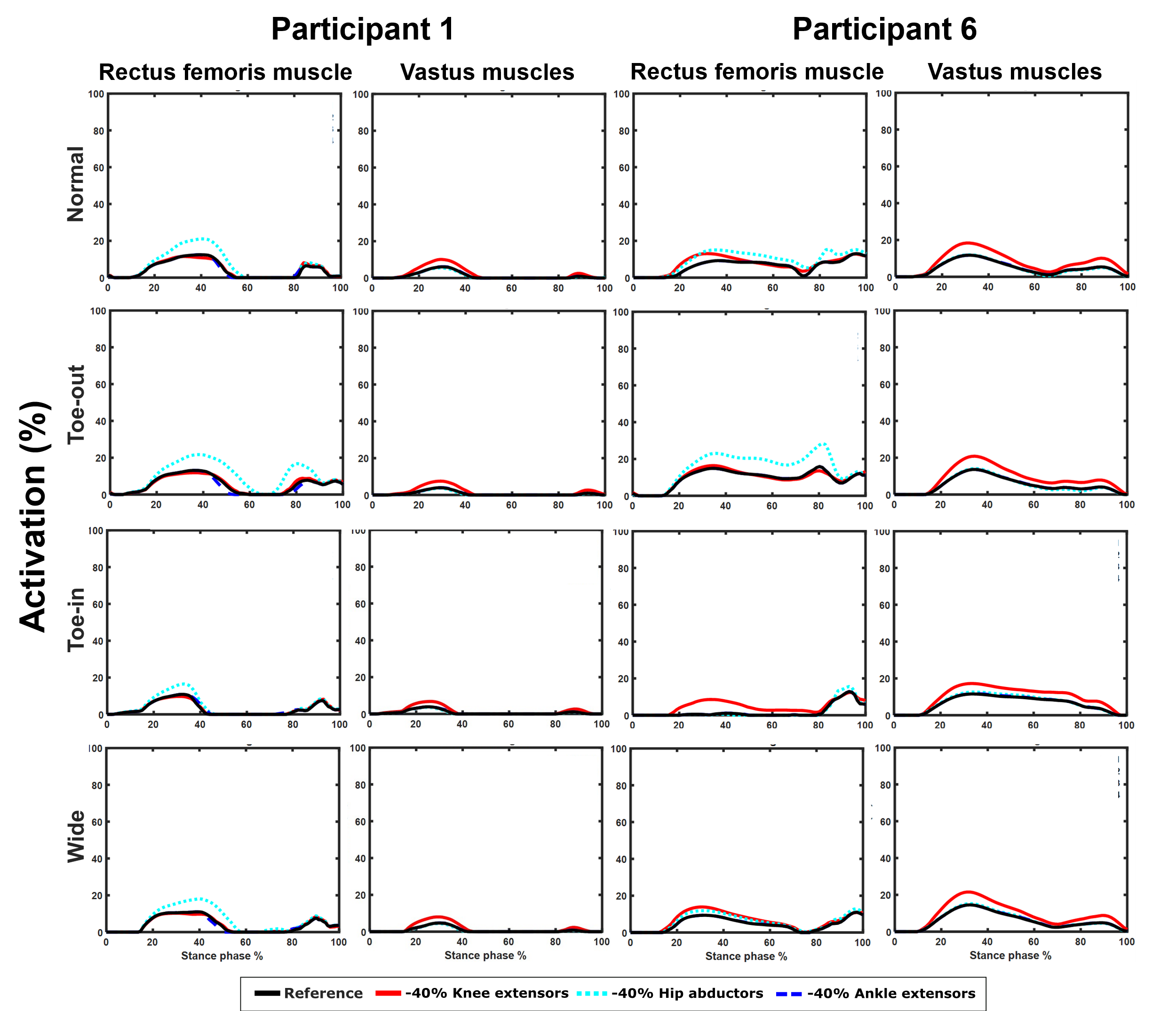


**Fig. S4**. Activations of gastrocnemius and soleus muscles in each gait style with all the muscle strength modifications for Participants 1 and 6. The reduced ankle extensor muscle strength led to increased activations of the soleus muscle in all gait styles. However, for Participant 1, in Toe-out gait the gastrocnemius muscle activation increased more than in the soleus muscle. In contrast, in Toe-in gait the soleus muscle activation increased more throughout the stance phase compared to the gastrocnemius muscle.


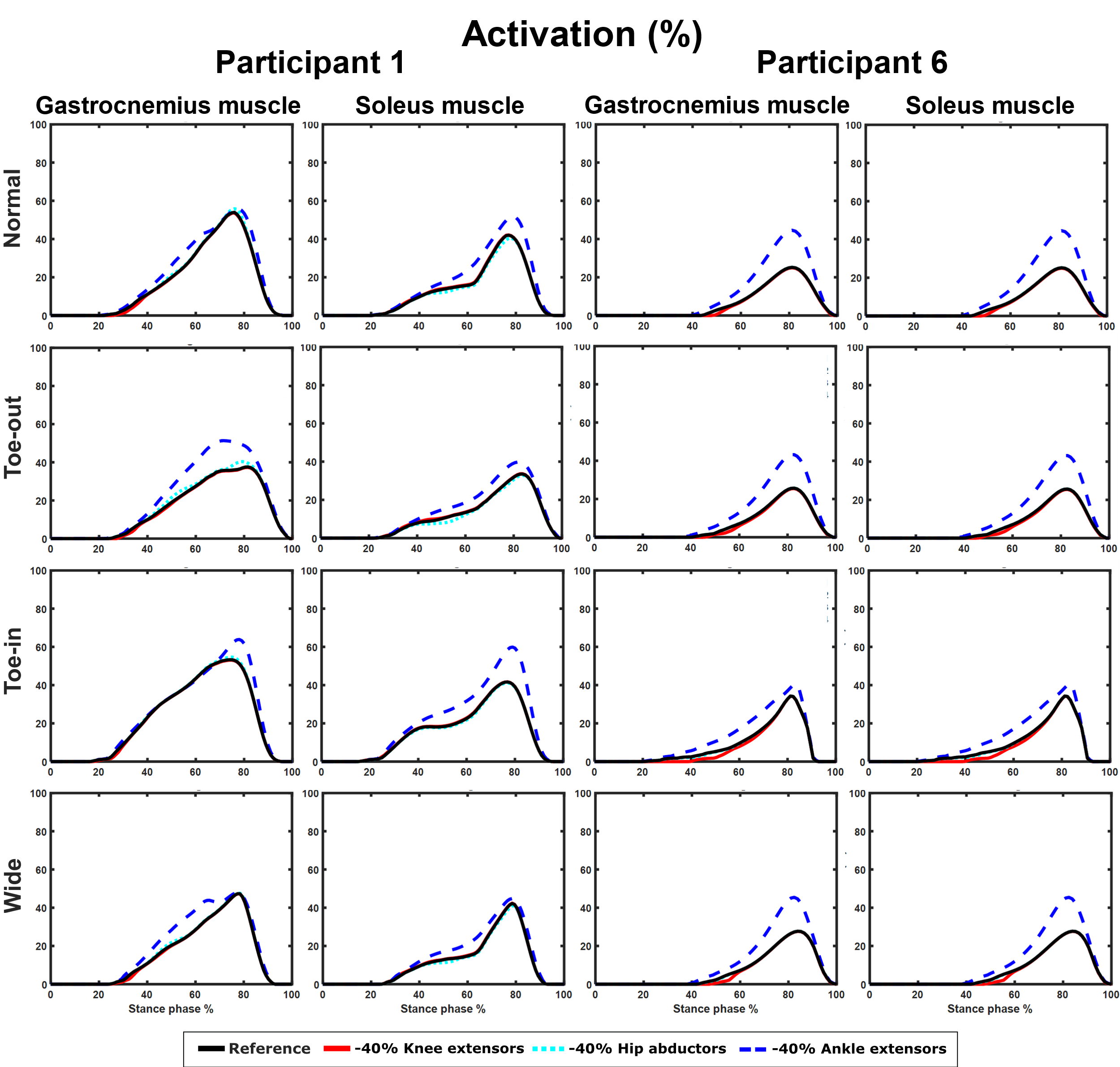


**REFERENCES**

1. Julkunen, P., Kiviranta, P., Wilson, W., Jurvelin, J.S., Korhonen, R.K.: Characterization of articular cartilage by combining microscopic analysis with a fibril-reinforced finite-element model. J. Biomech. 40, 1862–1870 (2007). https://doi.org/10.1016/j.jbiomech.2006.07.026

2. Dabiri, Y., Li, L.P.: Influences of the depth-dependent material inhomogeneity of articular cartilage on the fluid pressurization in the human knee. Med. Eng. Phys. 35, 1591–1598 (2013). https://doi.org/10.1016/j.medengphy.2013.05.005

3. Wilson, W., van Donkelaar, C.C., van Rietbergen, B., Ito, K., Huiskes, R.: Stresses in the local collagen network of articular cartilage: a poroviscoelastic fibril-reinforced finite element study. J. Biomech. 37, 357–366 (2004). https://doi.org/10.1016/S0021-9290(03)00267-7

4. Makris, E.A., Hadidi, P., Athanasiou, K.A.: The knee meniscus: Structure-function, pathophysiology, current repair techniques, and prospects for regeneration. Biomaterials. 32, 7411–7431 (2011). https://doi.org/10.1016/j.biomaterials.2011.06.037

5. Lipshitz, H., Etheredge, 3rd, Glimcher, M.J.: In vitro wear of articular cartilage. J. Bone Joint Surg. Am. 57, 527–534 (1975). https://doi.org/10.2106/00004623-197557040-00015

6. Esrafilian, A., Stenroth, L., Mononen, M.E., Tanska, P., Van Rossom, S., Lloyd, D.G., Jonkers, I., Korhonen, R.K.: 12 Degrees of Freedom Muscle Force Driven Fibril-Reinforced Poroviscoelastic Finite Element Model of the Knee Joint. IEEE Trans. Neural Syst. Rehabil. Eng. 29, 123–133 (2021). https://doi.org/10.1109/TNSRE.2020.3037411

7. Blankevoort, L., Huiskes, R.: Ligament-bone interaction in a three-dimensional model of the knee. J. Biomech. Eng. 113, 263–269 (1991). https://doi.org/10.1115/1.2894883

8. Butler, D.L., Kay, M.D., Stouffer, D.C.: Comparison of material properties in fascicle-bone units from human patellar tendon and knee ligaments. J. Biomech. 19, 425–432 (1986). https://doi.org/10.1016/0021-9290(86)90019-9

9. Atkinson, P., Atkinson, T., Huang, C., Doane, R.: A comparison of the mechanical and dimensional properties of the human medial and lateral patellofemoral ligaments. Proc. 46th Annu. Meet. Orthop. Res. Soc. Orlando, FL. (2000)

10. Villegas, D.F., Maes, J.A., Magee, S.D., Haut Donahue, T.L.: Failure properties and strain distribution analysis of meniscal attachments. J. Biomech. 40, 2655–2662 (2007). https://doi.org/10.1016/J.JBIOMECH.2007.01.015

11. Schatzmann, L., Brunner, P., Stäubli, H.U.: Effect of cyclic preconditioning on the tensile properties of human quadriceps tendons and patellar ligaments. Knee Surgery, Sport. Traumatol. Arthrosc. 6, S56–S61 (1998). https://doi.org/10.1007/S001670050224/METRICS
